# Pediatric Nephrolithiasis: A Changing Landscape Through Time and Space

**DOI:** 10.3390/medicina60121993

**Published:** 2024-12-02

**Authors:** Luca Pecoraro, Arianna Zuccato, Rebecca Vitella, Angelo Pietrobelli, Giorgio Piacentini, Milena Brugnara

**Affiliations:** Pediatric Unit, Department of Surgical Sciences, Destiny, Gynecology and Pediatrics, University of Verona, 37126 Verona, Italy

**Keywords:** pediatric nephrolithiasis, kidney stones, oxalate, calcium, uric acid, citrate

## Abstract

Pediatric nephrolithiasis is an ancient and complex disorder that has seen a significant rise in recent decades and the underlying causes contributing to stone formation in children may also be shifting. Historically, kidney stones have been linked to factors such as metabolic disorders, congenital abnormalities, and family history. However, the recent increase in incidence appears to be associated with new risk factors, including changes in lifestyle and diet, the growing prevalence of obesity, metabolic syndrome, diabetes, and even climate change. Given this evolving landscape, performing a comprehensive metabolic evaluation during the diagnostic process is essential. A complete metabolic evaluation should thus be performed during the diagnostic assessment to identify any modifiable risk factors predisposing to stone recurrence and reduce the need for surgical management, extrarenal comorbidity, and the increased burden of care.

## 1. Introduction

The rise in the incidence of kidney stones among children in recent decades has been a concerning trend, becoming an emerging public health concern. While the historical context of this disease stretches back centuries, the modern landscape of pediatric nephrolithiasis has shifted, with changes in lifestyle, diet, and environmental factors potentially contributing to the increasing prevalence. This condition imposes a significant economic burden, with costs related to diagnostics, treatments such as surgical interventions or lithotripsy, and long-term management strategies. Additionally, pediatric nephrolithiasis places a strain on healthcare systems, requiring specialized care and recurring follow-ups to prevent stone recurrence. Identifying the emerging risk factors behind this surge in pediatric stone disease is crucial, as it may inform prevention strategies and improve our understanding of this complex disorder. This review will explore the evolving epidemiology of kidney stones in the pediatric population, examining both the traditional and newly recognized risk factors [1,2]. The electronic databases Medline PubMed Advanced Search Builder, Scopus, and Web of Science were analyzed using the following medical subject headings (MeSH) terms and text words (as well as their combinations and truncated synonyms): “pediatric nephrolithiasis” “kidney stones”, “oxalate in pediatric nephrolithiasis”, “calcium in pediatric nephrolithiasis”, “uric acid in pediatric nephrolithiasis”, “phosphate in pediatric nephrolithiasis”, and “citrate in pediatric nephrolithiasis”. The abstracts were examined after the elimination of duplicate articles. The full text of relevant papers was analyzed. Studies providing the outcomes of case reports, case series, case-control studies, cohort studies, synthesized data, reviews, and randomized trials met the inclusion requirements. Only English-language articles were included in the research. The exclusion criteria included studies published only as abstracts, letters, conference proceedings, discussion papers, animal studies, and editorials. To find possibly relevant research, the titles were screened first, followed by the abstracts, and then, finally, the entire article was reviewed. Two reviewers (R.V. and A.Z.) independently evaluated all titles and abstracts. Quality assessments were performed by two independent reviewers (L.P. and A.P.); at the same time, the supervision was undertaken by the other authors (M.B., G.P., L.P.). All data were independently verified. After an initial search, an analysis based on the key questions and the inclusion and exclusion criteria narrowed down the results to 71 papers that met the inclusion criteria.

## 2. History and Epidemiology

The historical context of kidney stone disease stretches back thousands of years. Archaeological findings, such as examinations of Egyptian mummies, have revealed that kidney and bladder stones have afflicted humans since ancient times [1,2]. During the Middle Ages, the surgical removal of kidney and bladder stones was risky and mainly reserved for adults, as evidenced by archaeological findings and ancient texts like the Ebers Papyrus. Significant progress in understanding and treating the disease occurred in the 19th century [2,3,4]. In the pediatric population, the prevalence of kidney stones is notably lower compared to adults, with an estimated prevalence of about 1% [5]. However, over recent decades, there has been a noticeable increase in the incidence of nephrolithiasis in children, likely driven by factors such as changes in diet, lifestyle, and rising rates of childhood obesity. However, over recent decades, there has been a noticeable increase in the incidence of nephrolithiasis in children, likely driven by factors such as changes in diet, lifestyle, and rising rates of childhood obesity [5,6]. Data from adult populations in the US show that the incidence of kidney stones is higher in Western countries (Europe 5–9%, North America 12–15%) compared to Eastern regions (5%). Among individuals under 18, the incidence is estimated to be 5–10% of the adult rate [6]. In some areas of the Near and Middle East, as well as North Africa (e.g., Turkey, Saudi Arabia, Egypt, and Pakistan), nephrolithiasis is considered endemic, affecting 10–20% of the population. Several factors may explain these clusters, including the high consanguinity rates within these populations [6,7]. Furthermore, hot and dry climates and elevated ambient temperatures heighten the risk of dehydration, promoting stone formation. For example, in northeast Thailand, the high prevalence of renal tubular acidosis is linked to an increased rate of calcium oxalate stone formation and nephrocalcinosis [6,8]. Similarly, in some places like North India, Florida, and Ukraine, where the absence of *Oxalobacter formigenes,* an intestinal bacterium that degrades oxalate, is recognized, can lead to absorptive hyperoxaluria, which increases the risk of developing calcium oxalate stones [6]. Racial and ethnic differences in kidney stone prevalence have been observed for a long time. When comparing Third National Health and Nutrition Examination Survey (NHANES) II (1988–1994) with NHANES III (2007–2010), the increase in stone prevalence among Hispanics and African Americans was almost twice that of their white counterparts [9]. Population-based studies focused on the incidence of kidney stones in pediatric patients still need to be made available, with much of the available data limited to a few regional studies; these studies, conducted in countries such as Iceland, Japan, and the United States, provide valuable insights but still need to be comprehensive enough to capture the global burden of pediatric nephrolithiasis fully [6,10,11,12]. In Iceland, for example, a study reported an incidence of 5.6 cases per 100,000 children aged 0 to 18 years based on 26 newly diagnosed cases of nephrolithiasis over 6 years [10]. This research was conducted within a population of approximately 78,000 children, offering a small yet significant glimpse into the occurrence of kidney stones in this region. The relatively low incidence in Iceland may be linked to its specific environmental factors, dietary habits, or possibly genetic influences [10]. In contrast, a study from Japan revealed a notably higher incidence, particularly in the adolescent population [11]. By surveying hospitals and using diagnostic data from both imaging and clinical evaluations by urologists, researchers found the incidence of pediatric nephrolithiasis to be 17.7 per 100,000 in males and 12.4 per 100,000 in females, specifically within the age group of 10 to 19 years. These findings suggest potential gender differences in stone formation during adolescence, as well as variations in risk factors that could be regionally specific, such as dietary patterns, hydration practices, or even cultural aspects influencing health behaviors [6]. In the United States, data from South Carolina highlight a significant rise in the incidence of pediatric kidney stones over time. Pediatric emergency department visits revealed an increase in nephrolithiasis diagnoses from 7.9 per 100,000 children in 1996 to 18.5 per 100,000 in 2007 [12]. This sharp upward trend over just a decade underscores the evolving nature of the condition in modern pediatric populations. Another population-based study in the United States estimated the incidence of pediatric urolithiasis to be approximately 65 per 100,000 person-years between 2005 and 2016. This represents a significant rise compared to the 1999 estimate of 18 per 100,000 person-years [7,12]. The increase in stone formation among children is likely multifactorial, with contributing factors that may include changes in diet, such as increased consumption of processed foods, sugary drinks, and salt, as well as lifestyle changes leading to reduced physical activity and higher obesity rates. Environmental factors, such as rising temperatures and changes in hydration habits, may also play a role in this trend [13].

## 3. Kidney Stone Composition: A Changing Landscape?

The incidence and composition of urinary stones have significantly changed over the past 50 years, mostly in industrialized countries, reflecting profound shifts in living standards and dietary habits, with a dramatic increase in calcium oxalate stone cases [14]. A male predominance has been observed for calcium oxalate and uric acid stones, while calcium phosphate and struvite stones are more prevalent in females [14,15,16]. Between 1974 and 1977, stone analyses conducted by the Department of Urology at the University of Erlangen revealed consistent patterns in the frequency of different types of kidney and upper urinary tract stones. Calcium oxalate (CaOx) was identified as the most common stone type, followed by uric acid, calcium phosphate (CaP), struvite, and cystine [17]. Also, the composition of kidney stones in children has undergone significant and noteworthy changes, primarily driven by shifts in risk factors associated with diet, lifestyle, and other environmental influences [6]. The distribution of kidney stones in children now closely resembles that seen in adults, with current studies indicating that approximately 60–90% of these stones are composed of calcium oxalate, around 10–20% are made up of calcium phosphate, 5–10% are struvite stones, and less than 2–3% are uric acid stones [6,13]. This similarity in composition between pediatric and adult kidney stones highlights the pervasive nature of specific dietary and lifestyle factors across all age groups [6,13]. The prevalence of calcium oxalate stones has seen a noticeable increase among children [6]. This trend is probably linked to modern dietary patterns, with the growing availability and consumption of processed foods and increased sodium consumption [1,18]. These nutritional components can significantly contribute to the supersaturation of calcium oxalate in the urine, thereby promoting the formation of these stones [1]. On the other hand, the prevalence of struvite stones, which primarily develop due to bacterial urinary tract infections, has generally decreased over the years [1]. This decline is mainly due to improved hygiene practices, better access to medical care, and the effectiveness of antibiotics in treating and preventing recurrent urinary tract infections, which are the primary cause of struvite stone formation [1].

## 4. Known Risk Factors

### 4.1. Congenital Abnormalities of the Urinary Tract and Urinary Tract Infections

Anatomic or structural abnormalities, such as ureteropelvic junction obstruction, posterior urethral valves, duplicated system, bladder exstrophy, or prior lower urinary tract diversion, can lead to functional or obstructive issues, resulting in urinary stagnation [19]. Recurrent urinary tract infections, especially those caused by urease-producing bacteria (*Proteus*, *Klebsiella*, *Pseudomonas*), contribute to a higher urinary pH and the development of infection-related stones, such as struvite [20]. Urease breaks down urea into ammonia and bicarbonate, making the urine more alkaline. Magnesium ammonium phosphate and carbonate apatite can form in this alkaline environment, leading to stone formation. Struvite stones can grow quickly and form large staghorn calculi, underscoring the need for early diagnosis and management [21]. While urinary tract abnormalities and infections were previously major risk factors for stone development, better managing these conditions has made this type of stone less common in the pediatric population, now comprising only 5–10% of cases [22,23].

### 4.2. Family History

Kidney stone formation is a complex process, but research indicates that it often runs in families, with 35–60% of patients with stone disease having a positive family history [24]. Moreover, studies show that pathogenic, single-gene mutations account for a high proportion of kidney stone disease in childhood [25]. In an international cohort of 143 pediatric patients, mutations in 14 genes were detected, with younger patients more likely to have autosomal recessive conditions [26]. A causative mutation was identified in 17% of the cases, consistent with previous findings that around one in five pediatric patients with nephrolithiasis may have an underlying monogenic disorder [26]. Specific monogenic causes have been identified, including cystinuria, Dent disease, primary xanthinuria and primary hyperoxaluria [27]. Cystinuria accounts for 5–10% of pediatric nephrolithiasis cases, and at least two genes have been linked to this autosomal recessive amino acid transport defect [27]. While there is currently no clear indication for routine genetic testing, studies suggest that early genetic evaluation could reduce overall costs and time to diagnosis, especially with whole-exome sequencing (WES) [28]. Furthermore, a confirmed genetic diagnosis may enable more targeted and effective treatments, potentially reducing long-term healthcare expenditures [29].

### 4.3. Medications

Specific drugs are linked to an increased risk of kidney stone formation in children. These include loop diuretics, such as furosemide, which can promote hypercalciuria by inhibiting the reabsorption of calcium in the thick ascending limb of the loop of Henle [30]. Carbonic anhydrase inhibitors, such as acetazolamide, act in the proximal renal tubule by blocking the resorption of sodium bicarbonate. Consequently, these medications may induce a hyperchloremic metabolic acidosis, in which urinary pH increases and urinary citrate decreases, which can contribute to stone formation [31]. Antiepileptics, such as topiramate and zonisamide, have also been associated with kidney stones, likely due to a similar mechanism of action [32]. Moreover, ciprofloxacin and sulfonamides can contribute to kidney stone formation through distinct mechanisms: ciprofloxacin by crystallizing in alkaline urine, particularly at high doses, and sulfonamides due to their low solubility, especially in acidic urine, leading to crystalline aggregates [33]. The use of nephrotoxic drugs and diuretics during the neonatal period can promote the formation of kidney stones and nephrocalcinosis, especially in premature infants and those with low birth weight [34,35,36]. Later in life, chronic bowel diseases may become a risk factor due to increased intestinal absorption of oxalate [37]. In addition, the ketogenic diet, commonly used to treat epilepsy, has been particularly problematic, with incidence rates of kidney stones ranging from 3% to 6% over 2 years [34]. The use of potassium citrate has been recommended as a potential preventive measure against kidney stone formation in children following a ketogenic diet [38].

### 4.4. Metabolic Abnormalities

Among the various risk factors for kidney stone disease in children, metabolic abnormalities are the most prominent underlying drivers [39]. Hypercalciuria, a common metabolic risk factor, affects 30–60% of pediatric stone formers. It is defined as urinary calcium excretion exceeding 4 mg/kg in a 24-h sample and can have multiple potential causes in children [39]. Idiopathic hypercalciuria, which accounts for 50–60% of cases, arises from increased intestinal calcium absorption and decreased renal calcium reabsorption and often has a familial predisposition [40]. In this condition, the kidneys excrete excessive amounts of calcium, leading to urinary calcium supersaturation and an increased risk of stone formation [39,41]. To diagnose idiopathic hypercalciuria, excluding other conditions causing normocalcemic hypercalciuria, such as hereditary hypophosphatemic rickets with hypercalciuria, is crucial [39]. Less common secondary causes, including primary hyperparathyroidism, Cushing syndrome, and malignancies, may also contribute to hypercalciuria but are rare in the pediatric population [39,42]. Dietary factors, such as high sodium or protein intake, can exacerbate hypercalciuria in children. Notably, restricting dietary calcium should be avoided, as it may paradoxically increase calcium absorption, leading to bone demineralization and heightened oxalate absorption and excretion [39,43]. Instead, a diet low in salt, protein, and oxalate but adequate calcium is recommended to reduce the risk of stone formation and minimize lithogenic potential [43]. Hypocitraturia is another crucial metabolic disturbance in 20–60% of pediatric stone formers [44,45,46]. Citrate is an essential inhibitor of stone formation, as it binds to calcium and prevents the aggregation of calcium oxalate and calcium phosphate crystals. Hypocitraturia is defined as less than 365 mg/1.73 m^2^ in male children and less than 310 mg/1.73 m^2^ in female children in a 24-h urine sample [44,45]. Although hypocitraturia is usually idiopathic in origin, underlying conditions that cause metabolic acidosis and hypokalemia, such as distal renal tubular acidosis, should be considered [44,47]. Additionally, high-protein, low-alkali diets that emphasize animal protein and lack vegetable fiber and potassium can contribute to the development of hypocitraturia [44]. Addressing hypocitraturia through dietary modifications, such as increasing the intake of fruits and vegetables, can help reduce the risk of stone formation in pediatric patients [44,48]. Hyperoxaluria is also one of the leading metabolic disorders contributing to stone risk formation in the pediatric age. Recent studies show that hyperoxaluria affects approximately 20.6% of children diagnosed with nephrolithiasis [40,47]. Hyperoxaluria can arise from increased endogenous oxalate production in the liver or, more commonly, from factors that increase intestinal oxalate absorption or dietary intake [19]. The primary hyperoxalurias, types I and II, are rare autosomal recessive disorders caused by defects in specific hepatic enzymes that lead to the overproduction of oxalate. Secondary hyperoxaluria may be related to dietary factors, such as high oxalate-rich foods and low calcium intake [49,50]. Additionally, conditions that cause fat malabsorption, like short bowel syndrome, inflammatory bowel diseases and cystic fibrosis, can increase oxalate absorption and excretion [49]. Typical urinary oxalate concentrations in a 24-h urine collection are less than 45 mg/1.73 m^2^ for children of all ages, except for infants younger than one year, whose normal levels are below 13 mg/1.73 m^2^ [51]. Managing hyperoxaluria usually involves dietary modifications, such as limiting oxalate intake and ensuring adequate calcium consumption, to reduce urinary oxalate levels and the risk of stone formation [50,51]. Hyperuricosuria is another risk factor in 10–20% of pediatric stone formers and is defined as uric acid excretion of greater than 815 mg/dl/1.73 m^2^ of body surface area [52]. The most significant risk factor for the crystallization and formation of uric acid stones is low urinary pH, especially below 5.5, as this results in decreased solubility of uric acid [12]. Even if purely uric acid stones account for only 5% of stones in the pediatric population, however, hyperuricosuria may potentiate the formation of mixed calcium oxalate-uric acid stones, so it should be addressed in the overall management of pediatric nephrolithiasis [53]. Approximately 12% to 40% of children who have idiopathic hyperuricosuria have coexistent hypercalciuria [49]. Excessive endogenous production of uric acid can be caused by inherited defects of enzymes associated with purine metabolism, such as hypoxanthine-guanine phosphoribosyltransferase (HPRT) [54]. Hyperuricuria can be derived from dietary influences, particularly a high intake of purine-rich foods like red meat and seafood. Additionally, the rising prevalence of childhood obesity and metabolic syndrome, often linked to insulin resistance, can disrupt uric acid metabolism and contribute to hyperuricosuria [13]. Uricosuric drugs, renal tubular disorders, cyanotic congenital heart disease, hemolytic anemias and leukemias may cause hyperuricuria [49]. Dietary changes to reduce purine intake, coupled with pharmacological treatment if necessary, can help manage hyperuricosuria and lower the risk of uric acid and mixed stones in children [49]. Identifying children with an underlying disorder causing stone formation is essential, as these children have up to a 50% risk of recurrent stone formation, compared to less than 10% in those without an identifiable risk factor [55] (Table 1).

## 5. Current State of Pediatric Nephrolithiasis

The reasons for the increasing incidence of pediatric nephrolithiasis are unclear, but several potential contributing factors have been proposed. Multiple studies have explored the relationship between pediatric nephrolithiasis and factors like body mass index and obesity prevalence, driven by the increasing epidemic of childhood obesity and evidence from adult research connecting dietary and lifestyle factors to stone disease [6,51,56]. Increased body weight and higher body mass index in adults have been linked to elevated levels of calcium, oxalate, and uric acid in the urine and abnormally low urinary pH [5]. Despite these associations, recent epidemiological research describes conflicting results on whether a relationship exists in the pediatric population. David Sas et al. reviewed 155 children with stones and found that pediatric stone formers did not have higher BMI compared to the general pediatric population [57]. Moreover, Serica et al. noted that overweight children appear to have slightly increased pH compared to normal, suggesting that the relationship between BMI and urine pH may differ in children than adults [58]. On the other side, other studies have reported that children with obesity and metabolic syndrome are at greater risk of developing kidney stones. Tiwary et al. performed an observational study on 46 obese adolescents. They discovered that insulin-resistant obese patients exhibited an elevated relative saturation ratio of urinary calcium oxalate, suggesting an increased risk of calcium oxalate stone development. Furthermore, they noted that urinary pH decreased as metabolic syndrome risk factors increased. This is noteworthy because acidic urine promotes stone formation [58]. Specifically, insulin resistance can reduce urinary pH, leading to the development of uric acid stones [58]. Additionally, insulin resistance is associated with reduced citrate excretion, elevating the risk of calcium-based stones [58,59]. Kokorowski et al., performing a matched case-control study involving nearly 10,000 children and over 39,000 controls, conclude that pediatric urolithiasis is positively associated with obesity and hypertension but inversely associated with type I diabetes, suggesting that metabolic syndrome may play a role in the development of kidney stones in children [59]. Multiple other modifiable factors may also influence the risk of pediatric nephrolithiasis. Diet plays a significant role in the development of kidney stones in children. The role of diet is inherently connected to metabolic abnormalities, and many dietary factors may contribute to increased stone formation during childhood. Decreased fluid intake is an emerging risk factor, as changes in lifestyle and drinking habits have been observed worldwide [60]. Low fluid intake leads to reduced diuresis, resulting in concentrated urine; this may lead to the supersaturation of minerals, contributing to the formation of kidney stones [60]. An adequate fluid intake is the most important nutritional measure to prevent kidney stone recurrence, regardless of urine stone composition and individual risk factors for stone formation [60]. Multiple studies have found that increasing fluid intake to produce over 2.0–2.5 L of urine daily may reduce the risk of recurrent kidney stones [61]. Observational studies have suggested that increased fluid intake is a protective factor for primary stone prevention. According to the guidelines on urolithiasis, a copious fluid intake to maintain a urine volume of at least 2.0 to 2.5 L/24 h is recommended for most types of stones [51]. While randomized trials are still needed to conclusively demonstrate the efficacy of sustained hydration in preventing stone recurrence [19], a 2-year randomized trial is underway to investigate whether increased fluid consumption can effectively lower the risk of kidney stone recurrence in adults and children [51,62]. High sodium, protein and sugar intake have all been associated with an increased risk. At the same time, a diet low in citrate and high in oxalate and purines may also contribute to stone formation [61,63]. High intake of animal protein, salt, and high-fructose foods can lead to hypercalciuria, hyperoxaluria, and hyperuricosuria [61]. Specifically, the acid load provided by a high protein intake may increase urinary calcium and reduce urine pH and citrate excretion [61]. However, the relationship between protein intake and the risk of kidney stone formation remains a topic of ongoing debate, as highlighted by various systematic reviews and studies [63,64]. While the link between dietary protein and kidney stone risk remains unclear, extensive studies indicate that a higher net acid content in the diet is associated with an increased likelihood of developing kidney stones [19]. This suggests that the diet’s ratio of vegetables and fruits to protein may be a more reliable predictor of stone formation risk because the alkalizing effect of fruits and veggies can help neutralize the acid produced from consuming protein [19]. Furthermore, elevated sodium intake, consumed as sodium chloride (salt), can increase the amount of calcium excreted in urine. Sodium can interfere with calcium reabsorption in the renal tubules due to the increased volume of extracellular fluids, leading to hypercalciuria [65]. This surplus of urinary calcium can then be combined with other compounds, such as oxalate, increasing the risk of developing calcium-based kidney stones [19]. Still, some studies have questioned the direct relationship between dietary sodium and stone risk, as sodium may influence other stone risk factors like citrate and volume status [19]. Adequate calcium intake is important for reducing the risk of nephrolithiasis, as calcium can bind to oxalate in the intestines and prevent its absorption into the bloodstream, thereby reducing the availability of oxalate to form kidney stones [12]. Emerging evidence suggests a concerning link between climate change and the increasing incidence of urolithiasis, particularly rising global temperatures. The pathophysiological mechanisms behind this relationship are unclear, as is the specific role of climate elements like sunlight, temperature, and humidity [8]. However, it is hypothesized that dehydration and increased insensible fluid losses due to sweating may contribute to the increased supersaturation of lithogenic salts and the development of stones [66]. Numerous studies have documented that kidney stones occur more frequently in hotter areas, warm climates, or during the summer months. Furthermore, Tasian et al. found that as daily temperatures increased above 10 °C, the risk of kidney stone presentation over the following 20 days also increased in most cities [67]. This suggests an acute effect of temperature on stone formation. While no single temperature metric has emerged as the best predictor, recent work by Ross et al. indicates that moist heat metrics, which combine humidity and temperature (e.g., wet-bulb temperature, heat index), may more accurately predict kidney stone presentations than dry temperature alone [68]. This evidence has significant implications for the future burden of kidney stone disease, particularly given projected temperature increases due to climate change [8]. Recent findings have elucidated the relationship between antibiotic use and increased risk of kidney stones. Specifically, antibiotics may impact the intestinal microbiome, significantly regulating urinary oxalate levels [69]. The bacterium *Oxalobacter formigenes* helps degrade oxalate in the gut, reducing its absorption into the bloodstream and lowering urinary oxalate levels, thereby preventing the formation of calcium oxalate kidney stones [69]. However, certain antibiotics can disrupt the intestinal microbiome, including eliminating *O. formigenes* [69]. Without this oxalate-degrading bacterium, there is an increased risk of elevated urinary oxalate levels, contributing to kidney stone formation. This risk is particularly pronounced when antibiotics are used at younger ages and within 3 to 6 months before the diagnosis of nephrolithiasis [69]. These findings underscore the potential causal link between antibiotic use and kidney stone formation, highlighting the importance of antibiotic stewardship in healthcare [69]. Understanding the metabolic disorders that underpin pediatric nephrolithiasis underscores the critical need for targeted health policies aimed at prevention. These findings highlight the importance of early identification and management of modifiable risk factors, such as dietary habits, fluid intake, and underlying metabolic abnormalities. Developing evidence-based guidelines and promoting public health initiatives that emphasize prevention, education, and lifestyle modifications can significantly reduce the burden of kidney stones in children, improving long-term health outcomes and alleviating strain on healthcare systems. Table 2 summarizes established and emerging risk factors associated with pediatric nephrolithiasis.

## 6. Adapting Kidney Stone Prevention to Shifting Risk Factors

It is crucial to establish a custom-made framework for metabolic evaluation and provide clear guidance on treating and preventing kidney stones in children, given the significant risk of lifelong recurrence [19]. Identifying modifiable risk factors and underlying abnormalities is critical to reducing the likelihood of related complications, such as chronic kidney disease and the heightened risk of other associated health conditions [19]. Implementing strategies to reduce risk, such as dietary adjustments and specific pharmacological treatments, can profoundly prevent future stone formation and preserve long-term kidney function [19,62]. Maintaining sufficient fluid intake is the most crucial dietary strategy for preventing the recurrence of kidney stones, regardless of the type of stones or the individual’s specific risk factors for stone formation [19,60]. Adequate fluid intake should be regulated to maintain urine output above 2.5 liters per day, better if equally distributed throughout the day; urine output should exceed 750 mL per day for infants, 1000 mL per day for young children, and 2000 mL per day for children over 10 [62,70]. Avoiding sugar-sweetened soft drinks, especially those containing phosphoric acid, is essential because they are associated with higher serum uric acid levels and a greater frequency of hyperuricemia [19]. To prevent kidney stones, it is essential to incorporate a diet rich in fruits and vegetables. These foods naturally have a high water content, which helps increase urine volume. This increased urine output dilutes the concentration of substances that can form stones, making stone formation less likely [19,71]. Additionally, many fruits and vegetables are excellent sources of potassium and citrate. These nutrients inhibit the formation of calcium oxalate stones, the most common type of kidney stones [19]. They also help to make the urine more alkaline, reducing the concentration of stone-forming substances [71]. Limiting dietary sodium intake can decrease hypercalciuria. Adopting a low-sodium diet is recommended in cases of elevated urinary calcium excretion linked to high nutritional sodium (with urinary sodium levels exceeding 200 mmol/day) [19,43]. However, this can be challenging due to the prevalence of sodium in processed foods, where it is commonly added during manufacturing [18]. It is important to avoid restricting dietary calcium. Limiting calcium intake might seem like a good idea, but it can lead to increased intestinal absorption of oxalate, raising the risk of kidney stones. Children, in particular, should receive the recommended dietary allowance of calcium appropriate for their age to support healthy bone development [71]. While it is wise to avoid excessive protein consumption, since a high protein intake can lead to increased acid production in the body, causing more calcium to be excreted in the urine and reducing citrate levels, it is equally important not to restrict protein too much [64,71]. Protein is essential for growth, especially in children. The goal is to consume balanced protein to support healthy development without increasing the risk of kidney stones [71]. Daily calcium intake levels are generally recommended at 800 mg to 1 g, sodium between 2 to 3 g, and protein ranging from 0.8 to 1.4 g per kilogram of body weight. Additionally, limiting the consumption of foods high in oxalates to 40 to 50 mg daily is essential [51]. Citrus juices, like lemon, orange, and grapefruit, provide significant amounts of citric acid and may serve as alternatives to alkalizing medications [19]. A healthy diet and regular physical activity play a crucial role in preventing excess weight and metabolic syndrome, a condition that significantly increases the risk of developing kidney stones [19]. In a study conducted in Japan with 11,555 patients, it was found that individuals with more traits of metabolic syndrome had higher odds of developing kidney stones [11]. These metabolic traits were also strongly associated with increased urinary calcium, uric acid, oxalate, and lower citrate levels, contributing to kidney stone formation. The clustering of metabolic syndrome traits was linked to more severe kidney stone disease [11]. These findings suggest that kidney stone disease should be viewed as a systemic condition closely connected to metabolic syndrome [11]. Maintaining an appropriate Body Mass Index (BMI) for age is also crucial. Overweight or obese children were found to be more likely to have low urinary volume and elevated urinary uric acid levels than were normal weight-stone-forming children [5]. In summary, working on these modifiable risk factors and so encouraging balanced nutrition (adopting a balanced diet that includes plenty of fruits and vegetables, maintaining adequate calcium and vitamin D intake, and moderating protein consumption), regular physical activity, and healthy lifestyle habits from an early age, can help to minimize the risk of kidney stones.

## 7. Conclusions

Pediatric stone disease is a rapidly evolving field of study, and continued vigilance and expanded research into this concerning condition holds promise for reducing the disease burden across the lifespan. While genetic or systemic factors may contribute to some cases, these studies suggest that the increase in pediatric nephrolithiasis is more likely attributable to modifiable risk factors, like the growing obesity rates, metabolic syndrome, and changes in children’s diets. Addressing preventable causes, such as promoting healthy eating habits focused on a balanced diet with appropriate calcium, protein, and sodium intake, adequate hydration, and regular physical activity, is crucial to curbing the rising incidence of this condition in children. Pediatric nephrolithiasis is a multifactorial condition that necessitates a multidisciplinary approach involving nephrologists, nutritionists, pediatricians, and health educators. This integrated care framework is vital for developing effective prevention strategies and tailoring treatments to the unique needs of pediatric patients.

## Figures and Tables

**Table 1:** Summary of key metabolic disorders contributing to pediatric nephrolithiasis.

Metabolic Disorder	Definition	Prevalence	Causes	Management Strategies
Hypercalciuria	Urinary calcium excretion > 4 mg/kg in a 24-h sample	30–60% of cases	Idiopathic (50–60%), high intestinal calcium absorption, low renal reabsorption, familial predisposition; secondary causes: primary hyperparathyroidism, Cushing syndrome, malignancies, high sodium/protein intake.	A diet low in sodium, protein, and oxalate; adequate calcium intake; avoiding restricting calcium to prevent bone demineralization; pharmacological treatment for underlying causes if present.
Hypocitraturia	Citrate < 365 mg/1.73 m^2^ in males or <310 mg/1.73 m^2^ in females in 24-h urine	20–60% of cases	Idiopathic: underlying conditions like distal renal tubular acidosis, metabolic acidosis, hypokalemia; high-protein, low-alkali diets; low intake of fruits/vegetables.	Increase intake of fruits and vegetables; address metabolic acidosis; dietary changes to enhance citrate levels; potential use of citrate supplements if nutritional changes are insufficient.
Hyperoxaluria	Urinary oxalate > 45 mg/1.73 m^2^ (or >13 mg/1.73 m^2^ in infants)	~20.6% of cases	Primary (genetic enzyme defects); Secondary: high oxalate diet, low calcium intake, fat malabsorption (e.g., short bowel syndrome, cystic fibrosis, IBD).	Limit dietary oxalate intake; ensure adequate calcium consumption to reduce oxalate absorption; treat underlying conditions like malabsorption syndromes; consider pharmacological treatments for primary hyperoxaluria.
Hyperuricosuria	Uric acid >815 mg/1.73 m^2^ (<484 mmol) in 24-h urine	10–20% of cases	High purine diet (e.g., red meat, seafood); metabolic syndrome, insulin resistance; genetic enzyme defects; secondary causes: uricosuric drugs, renal tubular disorders, hemolytic anemia, leukemia.	Reduce dietary purine intake; weight management for obesity/metabolic syndrome; pharmacological treatments like allopurinol in severe cases; increase urine alkalinity to dissolve uric acid stones; treat underlying disorders.

**Table 2:** Established and emerging risk factors for pediatric nephrolithiasis.

Risk Factor Category	Specific Risk Factor	Notes	Type of Evidence
Dietary Habits	High Sodium Intake	There is an increase in calcium in urine, but there is some debate on the direct link to stones.	Established
	Low Fluid Intake	Concentrated urine increases the risk of crystal formation.	Established
	High Protein Intake	Increase in urinary calcium, reduction of citrate; ratio of protein to fruits/vegetables seems key.	Established
	High Oxalate Intake	Oxalate binds calcium, forming stones; found in spinach, chocolate, nuts.	Established
	High Purine Intake	Purines break down into uric acid, another stone former; found in red meat and organ meats	Established
	Low Calcium Intake	It is counterintuitive but can reduce oxalate binding in the gut, increasing the risk.	Established
Environmental Factors	Climate Change	Higher temperatures may increase dehydration, a key risk factor.	Emerging
Medical History	Antibiotic Use	Antibiotics may disrupt gut bacteria that break down oxalate, increasing the risk.	Emerging
Family History	Genetic Predisposition		Established

## Data Availability

No new data were created.
